# Exploring Factors Associated with Physical Exercise Participation Among Chinese Adults Based on Explainable Machine Learning Methods

**DOI:** 10.3390/bs16020233

**Published:** 2026-02-06

**Authors:** Tianci Lu, Baole Tao, Hanwen Chen, Jun Yan

**Affiliations:** College of Physical Education, Yangzhou University, Yangzhou 225127, China; dx120240094@stu.yzu.edu.cn (T.L.); dx120190064@yzu.edu.cn (B.T.); dx120230091@stu.yzu.edu.cn (H.C.)

**Keywords:** Chinese adults, physical exercise participation, China General Social Survey, machine learning

## Abstract

Background: Insufficient physical exercise is a growing public health concern in China, where only 30.3% of adults exercise regularly. Exploring the key factors associated with physical exercise participation is essential for promoting healthier lifestyles. Method: This study utilized data from the 2021 China General Social Survey (CGSS) to apply a progressive framework of dimensionality reduction, machine learning prediction, and SHAP-based interpretability analysis. A total of 19 potential factors were considered, with LassoCV used for feature selection and multiple models constructed for comparison. Results: The SVM model showed the best predictive performance. SHAP analysis revealed that watching sports events, household registration, educational attainment, subjective well-being, smoking, age, sleep quality, social activities, and residence suitability for physical exercise are the most important factors influencing participation. Higher education, greater subjective well-being, urban residency, frequent sports viewing, and residence suitability for physical exercise were positively associated with participation, while smoking and poor sleep quality were negatively associated with it. Conclusion: This study highlights the value of combining machine learning with interpretability methods to uncover the key predictors of physical exercise. The findings provide new evidence on the social, psychological, and environmental factors associated with Chinese adults’ exercise behavior, offering insights for targeted health promotion strategies.

## 1. Introduction

A large number of evidence-based studies have demonstrated that regular physical exercise is a key health behavior for preventing chronic diseases, improving mental health, and enhancing quality of life (Penedo & Dahn, 2005). However, insufficient physical exercise is a widespread public health challenge worldwide. According to the World Health Organization’s 2021 Global Health Report (WHO, 2021), more than 27% of adults do not meet the recommended physical exercise guidelines. Despite continued implementation of health promotion policies worldwide, improvements in this area over the past decade have fallen short of expected targets. As a country with a large population, only 30.3% of adults in China regularly participate in physical exercise (General Administration of Sport of China, 2022). This persistent lack of physical exercise participation can lead to physiological pathways such as energy metabolism imbalance and elevated inflammatory factor levels (Pinckard et al., 2019), exacerbating the burden of cardiovascular disease, type II diabetes, and obesity (Yan et al., 2013). It can also have indirect health effects through psychosocial mechanisms such as stress regulation disorders and impaired social functioning (Cohn-Schwartz, 2020). Therefore, it is of great practical significance to conduct a systematic analysis of the multidimensional factors underlying Chinese adults’ physical exercise participation.

Despite increasing research attention on the determinants of physical exercise, two key gaps remain. First, the proportion of Chinese adults who regularly participate in physical exercise has remained persistently low. Despite the introduction of multiple health promotion policies, the overall improvement has been limited. Second, previous studies have relied mainly on traditional regression-based statistical methods, which fall short in revealing the mechanisms of impact for complex, multi-layered, and potentially nonlinear relationships. In the Chinese context, there remains a lack of systematic research examining the multidimensional factors influencing adult physical exercise behavior using advanced computational methods, such as machine learning. Filling this gap is of great significance for both academic research and public health practice.

To construct machine learning models grounded in theoretical foundations, this study utilizes the social ecological model (SEM) and the theory of planned behavior (TPB) as the theoretical framework for variable selection and categorization, rather than for structural model construction. While the SEM provides a hierarchical perspective to identify external environmental constraints (macro and meso levels), TPB complements this by focusing on internal psychological drivers and behavioral readiness (micro level). Specifically, we mapped the available variables from the 2021 China General Social Survey (CGSS 2021) dataset onto the core constructs of these theories to ensure the multidimensionality of the predictive features.

At the micro-individual level (corresponding to TPB’s Perceived Behavioral Control and Attitudes), demographic and sociological characteristics influence individuals’ decision-making through resource acquisition capabilities, serving as proxy measures for Perceived Behavioral Control (PBC) (Metcalfe & Lindsey, 2020; Churilla et al., 2018; Eime et al., 2015). (Lee & Kang, 2024) employed machine learning methods to analyze the NHANES database and found that educational attainment is a significant enabler of participation decisions. Health cognition acts as an internal risk assessment mechanism, shaping attitudes towards promoting health through exercise (Edelstein, 2023). Similarly, perceived sleep quality regulates expectations of physical capability, further constraining or facilitating PBC (Cullen et al., 2019). Additionally, subjective well-being (S. Chen et al., 2021), perceiving social fairness (Sui et al., 2024), and social class (Yang et al., 2022) modulate internal psychological states relevant to behavioral intentions.

At the meso-environmental level (corresponding to TPB’s Subjective Norms), social capital and trust reflect the influence of social networks. Social capital reduces initiation costs through peer modeling (Z. Gao et al., 2024), while social trust enhances collective efficacy (Stevens et al., 2018). These factors create informal subjective norms, in which social pressure to continue exercising prevents discontinuation.

At the macro-structural level (SEM’s Environmental Context), the built environment serves as an external facilitator of PBC. The accessibility of sports facilities reduces time and energy costs (Prince et al., 2022), while community safety reduces perceived risks (Elshahat et al., 2020), thereby increasing the feasibility of exercise. Structural factors like household registration and occupational status reflect broader resource allocation systems that constrain individual opportunities (Wang & Zhou, 2018).

Furthermore, lifestyle habits interact with exercise behaviors: smoking and drinking may have a trade-off relationship with exercise (Mi-Ok et al., 2018; Barrett et al., 2023) (e.g., nicotine dependence reduces motivation), while positive behavioral changes (e.g., quitting) may promote participation (Nduaguba et al., 2019). Meanwhile, cultural norms (e.g., watching sports) reinforce subjective norms through implicit discipline (Ren & Liu, 2022). Details shown in Table 1.

However, traditional linear models often struggle to handle high-dimensional data and accurately capture the strength and direction of interactions between variables. In contrast, machine learning methods can efficiently process high-dimensional datasets, capture complex nonlinear relationships, and maintain high predictive performance. At the same time, interpretive tools can translate predictive outcomes into transparent and policy-relevant insights, thereby bridging the gap between predictive accuracy and interpretability. These methodological strengths make machine learning particularly well-suited for exploring the multidimensional and heterogeneous determinants of physical exercise participation. Moreover, machine learning is increasingly applied in behavioral and mental health research to support prediction, phenotyping, and decision-making through multidimensional behavioral and clinical data (Dwyer et al., 2018).

Based on the aforementioned theoretical framework, this study constructs a multidimensional predictive framework. We operationalized the theoretical constructs into four observable dimensions for feature engineering: (1) Demographic and Sociological Dimension (reflecting individual PBC resources: gender, age, educational attainment, household registration, BMI, socioeconomic status), (2) Psychological and Cognitive Dimension (reflecting attitudes and internal states: self-rated health, social trust, perceived social fairness, subjective well-being, perceived social class), (3) Lifestyle Dimension (reflecting behavioral trade-offs and norms: watching sports events, social activities, smoking, drinking, sleep quality), and (4) Built Environment Dimension (reflecting external PBC facilitators: residential area suitable for exercise, public facilities, community safety). Based on this, we propose the following core hypothesis: the physical exercise participation patterns of the adult population in China are associated with demographic and sociological characteristics, psychological cognitive schemas, lifestyle preferences, and characteristics of the built environment. To address the research gaps outlined above, this study adopts a progressive analytical framework of “regularization dimension reduction–predictive model optimization–interpretability conversion.” By compressing redundant variables, the framework enhances model robustness. A systematic algorithm balances predictive accuracy and interpretability compatibility. It also analyzes heterogeneous patterns of group-level influences on key predictive factors, aiming to provide a methodological reference for health behavior research that balances stability and interpretability.

## 2. Materials and Methods

### 2.1. Ethics Approval

The data used in this study came from publicly available anonymized datasets. All data are extracted from publicly available databases and have been anonymized before use, so they do not involve identifiable personal information. No specific ethical approval was required as the study was based solely on these anonymized, publicly available datasets.

### 2.2. Data Sources

The data for this study come from the CGSS 2021, which surveyed and analyzed 8148 Chinese residents. The China General Social Survey began in 2003 and is China’s earliest national, comprehensive, and continuous academic survey project. The data collected cover a wide range of social, community, family, and personal information. This paper organizes and cleans the CGSS 2021 database, which includes 22 variables (including the dependent variable physical exercise participation and height and weight variables used to calculate BMI), covering four major categories: demographics, individual subjective feelings, lifestyle, and built environment.

### 2.3. Research Subjects

**Figure 1:**
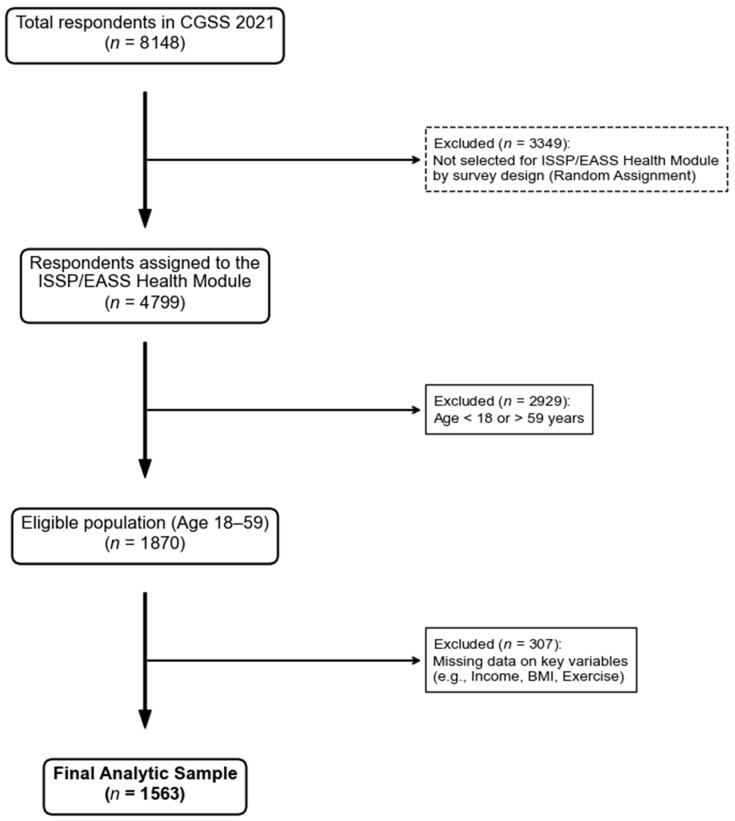
Variable screening process.

This study utilized data from the CGSS 2021, with an initial total of 8148 respondents. The CGSS 2021 randomly assigned respondents to different thematic modules. According to the survey design, the East Asian Social Survey (EASS) Health Module, the International Social Survey Programme (ISSP) Health Module, and the ISSP Environment Module were each administered to a randomly selected one-third of the respondents. Consequently, only respondents assigned to modules containing our variables of interest were eligible for this study (*n* = 4799). From this eligible subsample, we further applied the following exclusion criteria: (1) respondents outside the age range of 18–59 years (*n* = 2929 excluded); and (2) cases with missing data on key variables (*n* = 307 excluded). This resulted in a final analytic sample of 1563 participants. The detailed sample selection process is illustrated in Figure 1.

### 2.4. Variable Measurement and Data Processing

The purpose of this study is to explore the factors that influence physical exercise participation among Chinese adults. Therefore, the dependent variable of physical exercise participation was first selected, using item A30_9 (“In the past year, did you often participate in physical exercise during your free time?”), and reverse scoring was applied, with higher values indicating greater frequency of physical exercise participation. In terms of demographics, the gender question code (A2) was included and converted to a dichotomous variable, with male = 1 and female = 2 in the original table revalued as male = 1 and female = 0. Age (question code A3_1) was calculated using the year of the survey, 2021, and the respondent’s date of birth. Educational attainment (question code A7a) had with 14 options. For statistical convenience, this variable was recoded as follows: illiterate = 1; private tutoring/literacy classes = 2; elementary school = 3; junior high school = 4; vocational high school, regular high school, secondary vocational school, or technical school = 5; associate degree (adult higher education, university diploma (regular higher education), university bachelor’s degree (adult higher education), university bachelor’s degree (regular higher education) = 6; master’s degree and above = 7. Household Registration (question code A18): In this study, non-agricultural household registration, resident household registration (formerly agricultural household registration), and resident household registration (formerly non-agricultural household registration) were merged into resident household registration, while agricultural household registration remained unchanged. Military household registration and other categories were treated as missing values due to the small number of cases. The BMI is calculated using height and weight information as follows (WHO, 2000): BMI = weight (kg)/[height (m)]^2^. Socioeconomic status (item code A43e) is scored inversely, with higher values representing higher socioeconomic status. Subjectively, self-rated health (question code A15) uses raw data, with higher values indicating a more positive perception of one’s health status. Social trust (item code A33) uses raw data; higher values indicate higher levels of social trust. Perceiving social fairness (A35) uses raw data, with higher values indicating a greater sense of social fairness. Subjective well-being (item code A36) uses raw data, with higher values indicating higher levels of subjective well-being. Social class perception (item code A43a) uses raw data, with higher values indicating a higher perceived social class. In terms of lifestyle, watching sports events (item code A30_10) is scored in reverse, with higher values indicating more frequent viewing. Social activities (item code A31_1) use raw data, with higher values indicating more frequent social activities. Smoking (item code E18) is reverse-scored, with 1 indicating rarely/never smoking, 2 indicating a former smoker, and 3 indicating a current smoker. Alcohol consumption (item code E19) is reverse-scored, with higher values indicating higher frequency. Sleep quality (item code E24) is reverse-scored, with higher values indicating better sleep quality. In terms of the built environment, the residence suitable for physical exercise (question code E36_A) is measured using raw data, with higher values indicating greater suitability. Using the original data, a higher value indicates that the residential area has adequate public facilities (question code E36_C). Using the original data, regarding neighborhood safety, the higher the value, the safer the place of residence (question code E36_D). Variable selection and processing are detailed in Table 2.

### 2.5. Machine Learning Models

The machine learning method used in this study is based on the Python 3.8.18 kernel and implemented using Jupyter Notebook 7.0.8. Before starting model training, the dataset was split into 8:2, and 5-fold cross-validation was performed using the StratifiedKFold method to ensure the category distributions of both the training and test sets remained consistent with those of the original dataset. This method effectively avoids category imbalance issues and evaluates model performance across different data subsets, thereby providing an objective assessment of the model’s generalization ability. Given that the variables preliminarily selected in this paper that may be related to physical exercise participation are numerous, the LassoCV method was used to screen the features, thereby avoiding model overfitting and the influence of redundant features. LassoCV is a feature selection method that introduces L1 regularization and automatically selects the optimal regularization parameter via cross-validation over different regularization strengths. This study employed 5-fold cross-validation with LassoCV to more robustly evaluate the model’s performance across multiple training and validation sets, thereby enhancing the reliability of regularization parameter selection. Specifically, we denote the regularization strength by α. Prior to Lasso, all predictors were standardized to ensure that the L1 penalty is applied on the same scale. Candidate α values were set as 100 logarithmically spaced points from 10^−6^ to 10^1^. The optimal α (α_min) was selected using the minimum mean cross-validation error criterion, i.e., the α that minimized the mean cross-validated mean squared error (MSE) across folds. This method can not only effectively compress the coefficients of unimportant features but also shrink the coefficients of certain irrelevant or redundant features to zero, thereby screening out features that contribute significantly to the target variable while reducing the model’s dimension and improving its generalization ability. This method achieves a good balance between reducing complexity and maintaining model accuracy. After screening for important features using LassoCV, this study used five common machine learning models to model and compare, further verifying the impact of the screened features on the models’ predictive performance. These models include eXtreme Gradient Boosting (XGBoost), Light Gradient Boosting Machine (LightGBM), Random Forest, Gradient Boosting, and AdaBoost. These models employ ensemble methods, such as bagging and boosting, to comprehensively evaluate the performance of different algorithms on a specific dataset. Hyperparameter optimization was conducted using GridSearchCV with 5-fold cross-validation, and the scoring metric was set to ROC-AUC. To address the risk of overly coarse tuning, we expanded the search space using commonly recommended ranges and pilot runs. For XGBoost, the grid included n_estimators (100, 200, 300, 500, 800), learning_rate (0.01, 0.05, 0.1, 0.2), max_depth (2, 3, 4, 5), subsample (0.6, 0.8, 1.0), colsample_bytree (0.6, 0.8, 1.0), min_child_weight (1, 5, 10), reg_lambda (1, 5, 10), and gamma (0, 0.5, 1). For LightGBM, the grid consisted of n_estimators (100, 200, 300, 500, 800), learning_rate (0.01, 0.05, 0.1, 0.2), max_depth (−1, 2, 3, 4, 5, 7), num_leaves (15, 31, 63, 127), min_data_in_leaf (10, 30, 60), feature_fraction (0.6, 0.8, 1.0), bagging_fraction (0.6, 0.8, 1.0), bagging_freq (0, 1, 5), and lambda_l2 (0, 1, 5). For RandomForest, the grid comprised n_estimators (100, 200, 300, 500, 800), max_depth (None, 5, 10, 20), max_features (‘sqrt’, ‘log2’, 0.5, 1.0), min_samples_split (2, 5, 10), and min_samples_leaf (1, 2, 4). For GradientBoosting, the search space included n_estimators (100, 200, 300, 500, 800), learning_rate (0.01, 0.05, 0.1, 0.2), max_depth (2, 3, 4, 5), subsample (0.6, 0.8, 1.0), min_samples_split (2, 5, 10), and min_samples_leaf (1, 2, 4).

Five-fold cross-validation was used in the training process of the four machine learning models to effectively evaluate the robustness of the models and reduce the impact of data partitioning on model performance.

After completing model training and hyperparameter optimization, we implemented decision threshold optimization to enhance classification performance and overcome the limitation of using a fixed 0.50 threshold. Threshold selection was based on ROC curve analysis, and three optimization strategies were considered: First, sensitivity maximization under a specificity constraint: we searched all candidate thresholds and selected the threshold τ that maximizes sensitivity while maintaining specificity ≥0.70, i.e., τ = argmax{TPR(τ)|TNR(τ) ≥ 0.7}, where TPR denotes the true positive rate (sensitivity), and TNR denotes the true negative rate (specificity). Second, Youden index maximization, which identifies the threshold that maximizes J = TPR(τ) + TNR(τ) − 1. Third, F1-score optimization, which evaluates candidate thresholds to identify the one that maximizes F1 = 2 × Precision × Recall/(Precision + Recall). The threshold optimization procedure included (1) generating probability predictions for the test set using the trained model; (2) computing the ROC curve to obtain false positive rates, true positive rates, and the corresponding threshold sequence; (3) applying the selected optimization strategy to identify the optimal τ; and (4) reclassifying the test set using τ and calculating performance metrics including sensitivity, specificity, precision, F1 score, and accuracy.

By comparing the performance of each model under the same feature set (including metrics such as accuracy, precision, recall, F1 score, and AUC), the model with the best performance was ultimately selected for further analysis. To further explore the prediction mechanism of the optimal model, this paper introduces the SHAP (Shapley Additive Explanations) method to conduct interpretability analysis of the model’s predictions (Lundberg & Lee, 2017). The SHAP method is based on a game theory framework. It quantifies the importance of each feature and its direction of influence on the target variable by calculating each feature’s marginal contribution to the prediction output. Through this process, it is possible to obtain the specific mode of action of important features on the target variable. In addition to the SHAP summary plot, SHAP dependence plots were generated for the top predictors to visualize potential nonlinear, threshold, and saturation relationships between feature values and the model output. Smoking status was treated as an ordinal categorical feature (1 = never/rarely, 2 = former smoker, 3 = current smoker) and interpreted as discrete category-level effects in the dependence plot.

## 3. Results

### 3.1. Attrition Analysis

To evaluate potential selection bias, we compared the characteristics of the final analytic sample (*N* = 1563) with the excluded respondents (*N* = 6585) (Table 3). As expected, statistically significant differences were observed in the age variable. The average age of the included sample was significantly lower than that of the excluded sample (41.12 years vs. 54.14 years, *p* < 0.001), and the self-assessment of health was better. These differences stem directly from the study design, which deliberately excluded individuals aged 59 and older to focus on the adult population. However, no statistically significant differences were found between the included and excluded samples for key demographic variables, including gender (*p* > 0.05), household registration (*p* > 0.05), and BMI (*p* > 0.05). The stability of these core demographic characteristics indicates that sample attrition, primarily driven by the CGSS random sampling design, has not introduced systematic bias into the sample’s fundamental composition. Therefore, the observed differences reflect the specific characteristics of the target population in this study rather than non-random attrition due to data quality issues.

### 3.2. Variable Screening

**Figure 2:**
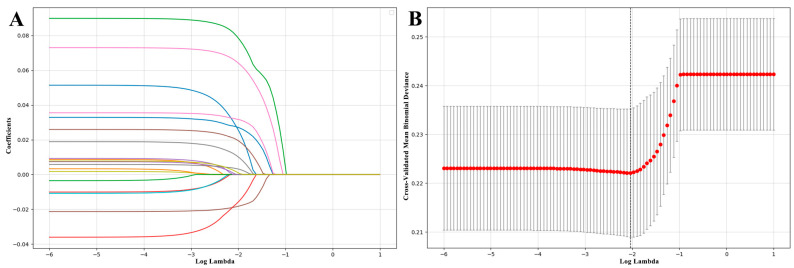
Variable selection based on LassoCV. (**A**) A path diagram showing how the regression coefficient changes with λ (regularization strength); (**B**) the process of determining the optimal λ through cross-validation, where the horizontal axis is log(λ) and the vertical axis is the cross-validated mean binomial deviance. Note: The red dots represent the mean cross-validation error for each λ value, and the error bars indicate ±1 standard error. The optimal λ is selected based on the minimum cross-validation error, corresponding to the most stable model performance.

This study builds upon previous research on the factors influencing physical exercise participation. After organizing and summarizing relevant data from the CGSS 2021 database, we initially identified 19 variables that may be associated with physical exercise participation: “age,” “gender,” “educational attainment,” “BMI,” “self-rated health,” “household registration,” “watching sports events,” “social activities,” “social trust,” “perceiving social fairness, “subjective well-being,” “perceived social class,” “socioeconomic status,” “smoking,” “drinking,” “sleep quality,” “residence suitable for physical exercise,” “residential area has sufficient public facilities,” and “neighborhood is very safe.” The LassoCV method was used to screen the variables in the training set, and the characteristics of the variable coefficient changes are shown in Figure 2A. This study used a 5-fold cross-validation method for iterative analysis to determine the optimal regularization parameter. Through analysis, nine variables closely related to physical exercise participation were selected, namely “age,” “educational attainment,” “household registration,” “watching sports events,” “social activities,” “subjective well-being,” “smoking,” “sleep quality,” and “residence suitable for physical exercise.” The regression coefficients of these variables are all non-zero, indicating that they make significant contributions to the target variable in the model.

### 3.3. Sensitivity Analysis

To address potential confounding bias and ensure robust feature selection, we specifically examined the 10 variables excluded by LassoCV. The complete comparison between bivariate associations and the full multivariate model is presented in Table 4. Bivariate analysis revealed that five of the excluded variables—self assessment of health, social trust, perception of social class, socioeconomic status, and the residential area has sufficient public facilities—were significantly associated with physical exercise participation (*p* < 0.05). However, in the sensitivity analysis, when fitting a full logistic regression model with all 19 predictors, none of these excluded variables retained statistical significance. This indicates that their effects were likely captured by the selected features due to multicollinearity. For instance, the effect of SES was likely mediated by educational attainment (Selected, *OR* = 1.38, *p* < 0.001), and the impact of the residential area having sufficient public facilities was likely redundant when paired with residence suitable for physical exercise (Selected, *OR* = 1.30, *p* = 0.01). In addition, regarding the specific concern about obesity, our analysis showed no significant association between BMI and exercise participation in either the bivariate (*p* = 0.23) or the multivariate sensitivity model (*p* = 0.45). This lack of significance justifies the Lasso algorithm’s exclusion of BMI in this dataset, distinguishing our findings from studies in which obesity serves as a primary correlate. Overall, the sensitivity analysis confirms that the LassoCV algorithm effectively filtered out redundant or non-significant predictors while retaining the most robust core variables.

### 3.4. Model Training and Performance Comparison

**Figure 3:**
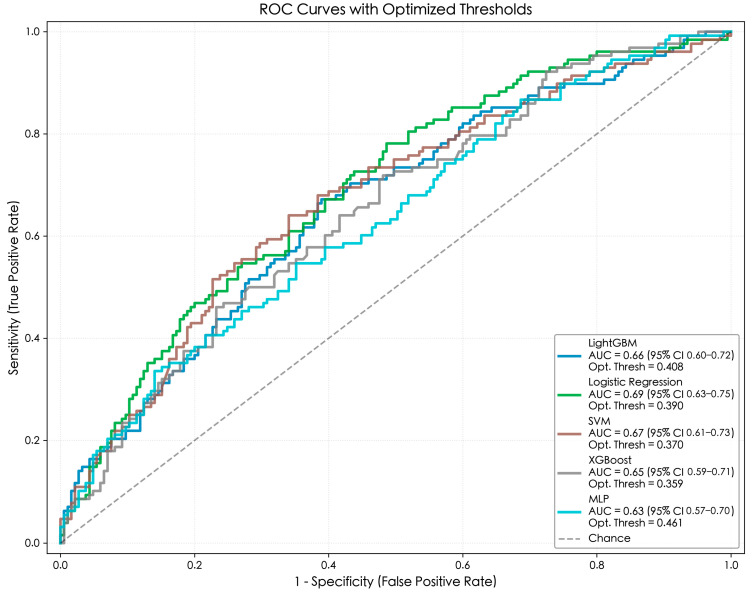
ROC curve of machine learning model.

After identifying factors closely associated with physical exercise participation, this study introduces logistic regression (LR) as a benchmark to rigorously evaluate the predictive utility of machine learning methods relative to standard benchmarks. Four machine learning algorithms are employed: SVM, LightGBM, XGBoost, and MLP. Table 5 presents detailed performance metrics, including comparisons between the default threshold (0.50) and the optimized threshold based on the Youden index. The corresponding ROC curve is shown in Figure 3. As shown by the ROC curve (Figure 3), the overall predictive discrimination of each model is moderate, with AUC values ranging from 0.63 to 0.69. The baseline linear model performed best in logistic regression (AUC 0.69, *95%CI*: 0.63–0.75), slightly outperforming support vector machines (0.67, *95%CI*: 0.61–0.73) and LightGBM (0.66). This indicates that the baseline linear model effectively captures the primary trends within the data. However, a detailed analysis of the threshold optimization metrics in Table 5 reveals key performance trade-offs, which serve as the basis for selecting the SVM over the baseline model. The optimized logistic regression model achieved the highest sensitivity (0.78) but had poor specificity (0.51), indicating a high false positive rate. This implies that linear models achieve high detection rates solely by aggressively classifying suspicious cases as exercisers, thereby limiting their actual precision. In contrast, the SVM model demonstrated the most robust and balanced performance after threshold optimization. By adjusting the decision threshold to 0.37, the SVM increased sensitivity to 0.64 (from 0.34 at the default threshold) while maintaining higher specificity (0.66). LightGBM (sensitivity 0.67, specificity 0.61) and XGBoost (sensitivity 0.72, specificity 0.52) also demonstrated improved detection capabilities under optimized thresholds, but failed to achieve the balanced discrimination performance of the SVM. Salganik et al. (2020) demonstrated that even complex models utilizing rich longitudinal data often yield limited predictive accuracy for multifaceted life outcomes, suggesting intrinsic limits to the predictability of human behavior in observational settings. Consequently, the current model performance substantially exceeds chance (AUC = 0.50) and is sufficient to identify key sociological determinants of exercise behavior.

Therefore, despite the slightly higher AUC value of logistic regression, this study still identifies the SVM as the optimal model. Its balanced trade-off between sensitivity and specificity makes it a more practical tool for policy interventions.

### 3.5. Interpretability Analysis

**Figure 4:**
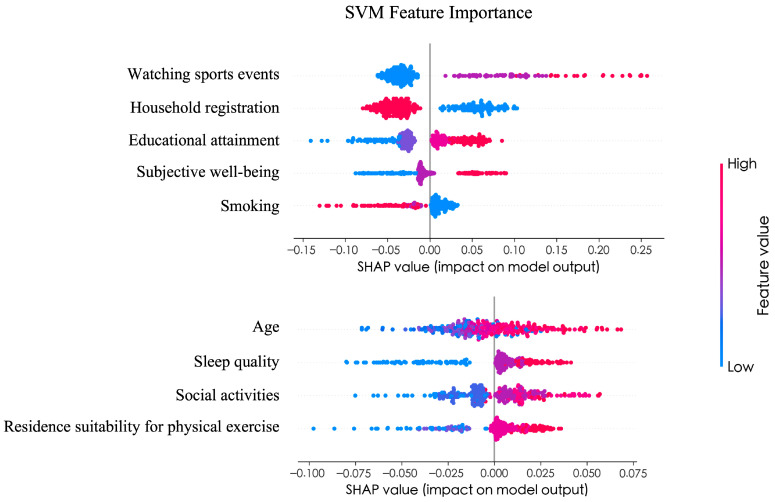
A SHAP summary plot for the SVM model. Note: SHAP values indicate the marginal contribution of each feature to the predicted probability of exercise participation; positive values increase the predicted likelihood of participation, whereas negative values decrease it. Color indicates the feature value (blue = low, red = high).

**Figure 5:**
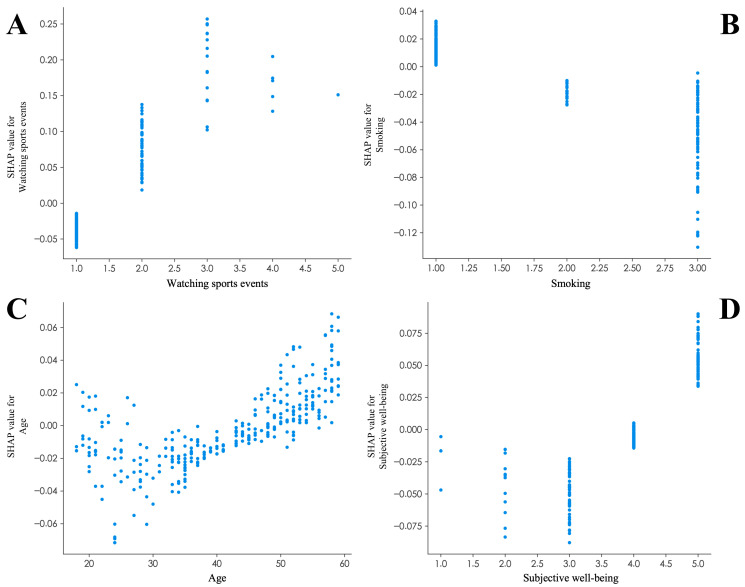
SHAP dependence plots for the top predictors in the SVM model. (**A**) Watching sports events, (**B**) subjective well-being, (**C**) age, and (**D**) smoking status. Note: Each point represents one respondent. Positive SHAP values indicate contributions that push the model output toward higher predicted exercise participation, whereas negative values indicate contributions toward lower predicted participation.

After determining that the SVM model has the best predictive performance, the Shapley Additive Explanations (SHAP) method was used to interpret the final model. As shown in Figure 4, the most important predictors of Chinese adults’ physical exercise participation were ranked as follows: watching sports events, household registration, educational attainment, subjective well-being, smoking, age, sleep quality, social activities, and residence suitability for physical exercise. From a feature distribution perspective, watching sports events had the largest mean absolute SHAP value, indicating the largest average contribution magnitude to model predictions. However, its practical significance should be interpreted in the context of its empirical distribution. As shown in Table 6, watching sports events was highly skewed toward the lowest category (median = 1, Q1–Q3 = 1–1), indicating limited variability for the majority of participants. For an interpretable probability-based context, increasing watching sports events from the minimum to the maximum observed level (1 to 5 on the 5-point scale) increased the predicted probability of exercise participation from 33.95% to 73.16%, holding other predictors at their sample means. Individuals who frequently watch sports events are more inclined to participate in physical exercise. Household registration and educational attainment also showed polarized patterns, with urban residency and higher education positively associated with participation. To further examine potential nonlinear relationships suggested by the SHAP summary plot, we generated SHAP dependence plots for the top predictors (Figure 5). Watching sports events showed a clear threshold pattern, with a marked positive shift from the lowest viewing level (1) to at least occasional viewing (≥2) and smaller marginal gains thereafter (Figure 5A). Subjective well-being exhibited a threshold effect. SHAP values were predominantly negative at levels 2–3, became close to zero around level 4, and turned distinctly positive only at the highest level (5) (Figure 5B). Age displayed a non-monotonic pattern, with more negative contributions in the late 20s–30s and increasingly positive contributions after midlife (Figure 5C). Smoking, coded as an ordinal categorical variable (1 = never/rarely, 2 = former smoker, 3 = current smoker), showed discrete stepwise differences: current smokers had the most negative SHAP values, whereas never smokers tended to have positive contributions (Figure 5D). Finally, lifestyle and the built environment also played meaningful roles; better sleep quality, more frequent social activities, and a residence suitable for physical exercise were generally associated with higher predicted participation.

## 4. Discussion

This study integrates behavioral theory with machine learning methods, offering a more systematic, data-driven perspective on the factors associated with physical exercise participation. By constructing a progressive analytical framework of “regularization dimension reduction–predictive model optimization–interpretability conversion,” this research systematically reveals the multiple driving mechanisms behind physical exercise participation among Chinese adults. Specifically, machine learning methods enable a more precise and quantitative characterization of individual factors influencing physical exercise, thereby overcoming the limitations of traditional statistical methods in handling complex, nonlinear relationships and multidimensional data. Furthermore, the analytical framework proposed in this study provides actionable theoretical and methodological references for future health behavior research based on predictive modeling. Based on the results, this study found that the key factors influencing Chinese adults’ physical exercise participation are in the following order of importance: “watching sports events”, “household registration”, “educational attainment”, “subjective well-being”, “smoking”, “age”, “sleep quality”, “social activities”, and “residence suitable for physical exercise”.

### 4.1. The Association Between Watching Sports Events and Physical Exercise Participation Among Chinese Adults

This study identified that the frequency of watching sports events was the strongest predictor of physical exercise participation among Chinese adults. However, given the cross-sectional design of this study, this result should be interpreted with caution regarding causality. The strong association likely reflects a complex, bidirectional relationship or shared underlying determinants rather than a simple one-way causal link where viewing drives participation.

First, reverse causality is highly plausible. Individuals who are already physically active are inherently more interested in sports and therefore more inclined to follow sports events as entertainment or to learn techniques. Second, watching sports events may function as a proxy variable for leisure time availability or cultural capital. Both regular exercise and watching sports require a significant investment of leisure time. Therefore, this variable also captures the subject’s underlying time affluence rather than a direct psychological motivation.

Importantly, the SHAP dependence plot suggests a threshold pattern (Figure 5A); compared with respondents who never watch sports events (level 1), even occasional viewing (level ≥ 2) is associated with a clear positive shift in SHAP contributions, whereas further increases in viewing frequency yield smaller marginal gains.

Despite these confounding factors, the potential reinforcing effect of watching sports on exercise behavior should not be ignored. The observational learning mechanism in social cognitive theory can explain this phenomenon. This mechanism emphasizes that human learning occurs not only through direct experience but also through observing others’ behavior and its outcomes (Jiang, 2024). Individuals can be inspired to participate in physical exercise by watching sports events, which can stimulate their interest and motivation. Ge and Dong (2019) suggest that spectator sports, such as football and basketball, can enrich the public’s perception of sports and, to a certain extent, enhance individuals’ motivation to exercise. Additionally, the rise of new media has facilitated convenient access to sports content, further stimulating viewers’ motivation to participate in sports (Bi & Huang, 2020). In addition, frequent viewing of sports events can expose individuals to the positive effects of athletic skill demonstrations and emotional encouragement, thereby reducing the cognitive load associated with athletic skills, enhancing confidence in one’s abilities, and forming a sense of self-efficacy that “I can accomplish similar athletic feats” (G. L. Zhang, 2015), ultimately correlating with higher physical exercise participation. In the Chinese context, this synergy is further amplified by the collective identity embodied in major national competitions (e.g., the Olympics or Asian Games), which often ignite public enthusiasm for fitness. Policy-wise, while we cannot claim that simply encouraging people to watch sports will directly lead them to exercise, the strong predictive value of this variable suggests that sports audiences are a prime target group for health interventions. Promoting the health benefits of physical exercise during major sporting broadcasts and organizing complementary activities, such as “nationwide workout sessions,” can effectively leverage this high-affinity audience, facilitating the transition from “spectators” to “participants.”

### 4.2. The Association Between Household Registration and Physical Exercise Participation Among Chinese Adults

This study found that respondents with urban household registration (resident hukou) have a higher predicted probability of reporting physical exercise participation than those with rural household registration (agricultural hukou). This pattern can be interpreted through the built environment dimension of the social ecological model. In the context of China’s urbanization, urban residents generally have more sports resources and organized opportunities to engage in exercise than rural residents (Ma et al., 2014). Meanwhile, migrant workers in cities tend to live in non-core areas where public sports facilities are often limited. As migrants, they face disadvantages in urban integration and social network building, which may further restrict access to sports resources through social ties (Peng, 2019). Differences in urban and rural lifestyles and cultural traditions may also contribute to divergent leisure patterns and exercise norms (Liu, 2002; Zhu & Zhang, 2014). More broadly, hukou is not only a residential classification but also an institutional status linked to unequal access to education, healthcare, employment, and public services—including fitness-related infrastructure—thereby shaping exercise opportunities across the life course. However, the observed hukou gradient should be interpreted with greater nuance because our outcome captures “physical exercise during free time” rather than total physical exercise. Rural adults can accrue substantial energy expenditure through agricultural labor, other physically demanding work, and domestic tasks, which could reduce both the perceived need for and the available time for recreational exercise. International evidence similarly suggests that rural–urban differences can appear larger when physical exercise is measured only during leisure time, because rural residents can accumulate activity through occupational or household domains (Whitfield et al., 2019; Pelletier et al., 2023). In addition, the wording of the CGSS item (“physical exercise during your free time”) can introduce response bias if respondents interpret “exercise” as a formal or recreational activity. Rural residents might be less likely to classify farm work or manual labor as “exercise” despite metabolic equivalence, which could lead to differential under-reporting of activity among agricultural hukou holders. Therefore, our results are best interpreted as an inequality in leisure-time exercise participation and related opportunities, rather than a definitive rural–urban gap in overall physical exercise levels. Although the hukou system is specific to China, the broader phenomenon of rural–urban disparities in leisure-time exercise has been documented internationally. For example, analyses of the U.S. National Health Interview Survey show that adults in nonmetropolitan counties are less likely than those in large metropolitan areas to meet aerobic and muscle-strengthening physical exercise guidelines during leisure time, highlighting the role of structural capacity and policy, systems, and environmental supports in rural settings (Abildso et al., 2023). These international comparisons suggest that, beyond the China-specific institutional mechanisms of hukou, unequal built environments and resource allocation can systematically shape rural–urban exercise disparities.

### 4.3. The Association Between Educational Attainment and Physical Exercise Participation Among Chinese Adults

This study found that educational attainment shows a polarized association with physical exercise participation among Chinese adults, validating the interaction between the social ecological model’s resource acquisition capacity and the cognitive assessment mechanism in the theory of planned behavior. First, individuals with higher education have a more substantial understanding of health knowledge due to their systematic educational background. They can accurately assess the long-term health benefits of exercise and effectively obtain scientific guidance on exercise, thereby achieving sustained participation. In contrast, low-educated groups tend to overestimate the risks of exercise or underestimate its benefits due to a lack of systematic health education, which directly weakens their motivation to participate (X. T. Li et al., 2024). Secondly, educational attainment remained a robust predictor of health decision-making ability even after controlling for multicollinearity via regularization-based dimensionality reduction. This suggests that educational attainment itself may independently predict individuals’ health decision-making ability through differences in the channels of knowledge acquisition. Higher educational attainment is associated with stronger abilities to process health information and develop exercise plans. Therefore, governments and communities should prioritize low-educated populations with relatively scarce health education resources when formulating public health policies. They should take the lead in developing accessible health education materials through diverse channels, such as short videos, community outreach sessions, and illustrated, science-based handbooks. This approach aims to break down cognitive barriers and correct the misconception that physical exercise carries “high risks and low benefits”.

### 4.4. The Association Between Subjective Well-Being and Physical Exercise Participation Among Chinese Adults

Currently, few scholars have explored the role of subjective well-being in physical exercise participation. This study found that higher subjective well-being was associated with higher predicted physical exercise participation. This phenomenon may be because positive emotions can expand an individual’s cognitive range and flexibility, thereby enhancing sensitivity to pleasurable experiences in a physical exercise context (e.g., mind–body feedback associated with endorphin release) (Y. J. Zhang & Cui, 2012), which in turn can form intrinsic motivation driven by emotional reinforcement. In addition, higher subjective well-being can lead to greater self-regulation efficacy, thereby reducing willpower depletion during the maintenance of exercise habits (Y. J. Zhang & Cui, 2012). The SHAP dependence plot provides evidence of a threshold effect (Figure 5B): SHAP values were predominantly negative at low-to-moderate levels (2–3), became close to zero around level 4, and turned distinctly positive only at the highest level (5). This suggests that moderate happiness may be insufficient to sustain behavioral motivation, whereas very high well-being can provide psychological resources that facilitate exercise adherence (Y. H. Chen & Yang, 2024). Given this finding, government or community advocacy for physical exercise should shift from the traditional “disease prevention” framing to a more positive “pursuit of joy” framing. During advocacy efforts, greater emphasis can be placed on the pleasure, sense of accomplishment, and physical and mental well-being that exercise provides. Physical exercise should be positioned as a lifestyle choice that enhances quality of life and overall happiness.

### 4.5. The Association Between Smoking and Physical Exercise Participation Among Chinese Adults

Smoking status was negatively associated with the predicted probability of engaging in leisure-time physical exercise. In our SHAP dependence plot, current smokers (coded as 3) showed the most negative SHAP contributions, whereas never/rarely smokers (coded as 1) tended to show positive contributions (Figure 5D). This pattern is broadly consistent with prior evidence linking smoking to impaired cardiorespiratory function and greater perceived exertion, which may reduce engagement in recreational exercise (Dugral & Balkanci, 2019; Y. L. Zhang et al., 2017). Smoking has also been associated with less adaptive stress coping styles, which can further correlate with lower motivation for physical exercise (Feng, 2016). Nevertheless, this association should not be interpreted as a one-way causal effect, because our analysis is cross-sectional. Reverse causation is plausible; individuals who decide to become more physically active may reduce or quit smoking first as part of a broader lifestyle change. In addition, selection effects and residual confounding may exist, as more health-conscious individuals are likely to both avoid smoking and seek active lifestyles. The “former smoker” category is also heterogeneous and potentially self-selected (e.g., long-term quitters with high health motivation, recent quitters, or individuals who quit due to illness). Therefore, the comparatively less negative SHAP contributions among former smokers may reflect differences in health motivation and quitting trajectories rather than a direct benefit of cessation captured by our data. Prior work suggests that physical exercise may complement smoking cessation efforts (e.g., by reducing cravings or increasing cessation-related self-efficacy) (Han & Zheng, 2018; Z. T. Zhang et al., 2024). However, receptor-level neurobiological explanations (e.g., dopaminergic pathway or receptor hypotheses) cannot be evaluated in the CGSS and should be considered speculative in light of our findings. From a public health perspective, smoking status can help identify groups with lower predicted exercise participation who may benefit from integrated programs that combine physical exercise promotion with cessation support. Future longitudinal or intervention studies are needed to disentangle bidirectional effects and examine how transitions between current, former, and never-smoking status relate to changes in exercise behavior.

### 4.6. The Association Between Age and Physical Exercise Participation Among Chinese Adults

The SHAP dependence plot suggests that the association between age and participation is nonlinear rather than strictly monotonic (Figure 5C). Predicted participation tends to be less favored (more negative SHAP contributions) during early-to-mid adulthood (approximately the late 20s to 30s), while the contribution of age becomes progressively more positive after midlife (around the 40s), indicating a potential life-course turning point. This pattern may reflect changing constraints and motivations across adulthood; individuals in their 20s–30s may face stronger time and energy constraints due to career development and family responsibilities (Ma et al., 2014), whereas midlife adults may place greater emphasis on chronic disease prevention and health maintenance, potentially supported by more stable routines and resources (X. Gao & Meng, 2013). Regarding the impact of age on individual physical exercise participation, policies should implement differentiated, lifecycle-based promotional strategies. For younger and midlife working-age adults, promotional efforts should emphasize the immediate benefits of exercise in alleviating work stress and boosting energy levels, and support flexible, time-efficient activity options. For later-midlife adults, the focus can shift to highlighting the long-term value of exercise in preventing chronic diseases and maintaining physical function.

### 4.7. The Association Between Sleep Quality and Physical Exercise Participation Among Chinese Adults

Poor sleep quality is significantly associated with lower physical exercise participation among Chinese adults. This phenomenon can be explained from both physiological and psychological perspectives. First, on a physiological level, deep sleep is crucial for tissue growth and regeneration. Research suggests that insufficient deep sleep can lead to reduced growth hormone secretion, which may impair the repair of muscle fibers, prolonging the recovery period after exercise and potentially affecting athletic performance (Zaffanello et al., 2024). Additionally, slow-wave sleep can lead to metabolic disorders in the frontal cortex, weakening an individual’s executive function and making it more challenging for them to execute predetermined plans during physical exercise. Secondly, on a psychological level, poor sleep quality can lead to decreased energy levels and worsened emotional states, which are linked to increased anxiety and depression and lower motivation and ability to participate in physical exercise.

### 4.8. The Association Between Social Activities and Physical Exercise Participation Among Chinese Adults

Social activities are positively associated with physical exercise participation among Chinese adults. This finding is consistent with many academic studies. It is generally believed that social activities provide opportunities for interaction with others, and group activities can give participants a stronger sense of social connection, offer opportunities for group exercise, and motivate individuals to engage in physical exercise. Research has found that, compared to people who do not frequently participate in social activities, those who regularly engage in social activities are significantly more active in terms of exercise frequency and persistence (Y. Li & Lv, 2023). Additionally, the positive correlation between social activities and physical exercise participation may stem from behavioral imitation and resource-sharing mechanisms among social network nodes. Specifically, strong social ties may reinforce individuals’ perceptions of health norms through peer pressure (e.g., mutual supervision of exercise check-ins) and demonstration effects (such as WeChat exercise step counts or exercise sharing on Moments), thereby promoting physical exercise participation (Wu et al., 2024). Weakly connected social networks, on the other hand, may utilize fitness apps to pinpoint users’ interests and needs with precision, thereby creating channels of communication between users. In this process, individuals receive heterogeneous information (e.g., on different sports), which lowers the cognitive threshold for exploring healthy behaviors and promotes physical exercise participation (Sun et al., 2024).

### 4.9. The Association Between the Built Environment and Physical Exercise Participation Among Chinese Adults

This study found that whether a place is suitable for physical exercise is significantly correlated with the frequency with which individuals participate in physical exercise. This finding can be explained from two perspectives: the built environment dimension of the social ecological model and the perceived behavioral control aspect of the theory of planned behavior. First, the social ecological model emphasizes the critical role of environmental factors in shaping individual behavior, particularly the impact of the built environment on physical exercise participation (Huang & Jin, 2023). The direct environment in which people live and the community’s provision of sports facilities directly determine residents’ access to and convenience of exercise. Guo Wen and other scholars found that the community environment has a significant positive association with residents’ exercise habits, including improvements in sports facilities and the diversity of sports activities (Guo et al., 2023). Secondly, the perceived behavioral control mechanism in the theory of planned behavior suggests that individuals’ sense of control over their behavior (including perceptions of the environment) influences their behavioral decisions (Guo et al., 2023). When an individual perceives that their living environment is suitable for physical exercise, they are more likely to believe they are capable and willing to engage in it. This perception of behavioral control is influenced not only by objective environmental factors but also by individual subjective cognition. This finding suggests that “exercise-friendly” residential environments are strongly linked to individual physical exercise participation. This necessitates that community planning and urban design prioritize safeguarding and upgrading public sports spaces—such as constructing neighborhood jogging paths, adding outdoor fitness equipment, and ensuring the accessibility and maintenance of facilities like sports courts. Currently, China’s government-led “National Fitness” initiative, led by the State Council of the People’s Republic of China (2021), has resulted in the widespread installation of fitness equipment in residential communities and parks, with its coverage continuing to expand.

## 5. Conclusions and Limitations

### 5.1. Conclusions

This study found that watching sports events is the most important predictor of physical exercise participation. The SHAP dependence plot suggests a threshold pattern, where moving from never watching to at least occasional watching corresponds to a clear positive shift in predicted participation. Household registration also plays a crucial role, with urban residents participating in physical exercise at a much higher rate than rural residents due to improved facilities and easier access to information. In terms of educational attainment, individuals with higher levels of education are more likely to adhere to exercise routines, possibly because they have greater health knowledge and are better at developing scientifically informed exercise plans. Subjective well-being also shows a meaningful association; very high subjective well-being contributes positively to predicted participation, whereas low-to-moderate levels show limited or negative contributions, consistent with a threshold effect. Smoking shows a clear negative association with physical exercise; current smokers are less likely to participate, while former smokers tend to have less negative contributions. The association with age is nonlinear, with lower predicted participation in early-to-mid adulthood and a more positive contribution after midlife. Finally, lifestyle and social factors also play a role. Better sleep quality is positively associated with participation, as poor sleep quality can undermine exercise intention and capacity both physiologically and psychologically. Social activities provide additional motivation, since socially active people are more likely to sustain exercise through mutual encouragement and group activities. The living environment is also relevant, as having suitable places for physical exercise in the community is associated with a higher frequency of participation.

### 5.2. Limitations and Future Research

This study has made certain progress in theoretical and empirical analysis, but several limitations remain. First and foremost, regarding outcome measurement, our reliance on a single self-reported item from the CGSS to gauge physical exercise constitutes a measurement constraint. Unlike objective measures derived from wearable devices (e.g., accelerometers), self-reported data rely on subjective recall, which may be prone to memory errors. Furthermore, given the normative nature of “exercise” as a healthy behavior, responses may be subject to social desirability bias, in which participants may unconsciously overreport their physical exercise levels to align with social expectations.

Second, regarding model performance interpretation, the modest predictive power (AUC = 0.67) suggests that unmeasured factors likely play important roles beyond the sociodemographic variables captured in the CGSS. Physical exercise is a complex behavior influenced not only by stable individual traits but also by highly dynamic contextual variables (e.g., weather conditions, accessibility of sports facilities) and momentary states (e.g., daily stress, immediate physical fatigue). Furthermore, the built environment was assessed using a limited set of items (only three indicators available in the survey). This coarse granularity fails to capture the multidimensionality of urban planning (e.g., green space density, street connectivity), which critically influences walkability and physical exercise. These unmeasured variances pose an intrinsic challenge for behavioral prediction and limit the model’s upper bound on precision.

Third, regarding feature selection, variables excluded via regularization (LassoCV) may, in theory, act as confounders. For example, while obesity is often linked to exercise, our sensitivity analysis revealed that BMI was not a significant predictor in this specific sample, justifying its exclusion. Similarly, variables such as socioeconomic status (SES) were likely excluded due to strong multicollinearity with educational attainment. Although our sensitivity analysis confirmed that the core predictors remained robust when these variables were included, the algorithmic exclusion of potential theoretical confounders remains a methodological limitation that future causal inference studies should address by strictly controlling for these factors. Crucially, the cross-sectional nature of the current study precludes the determination of directionality. This limitation is particularly relevant for lifestyle behaviors such as smoking and exercise, for which reverse causation and self-selection (e.g., health-motivated quitters) are plausible. Future research should therefore employ longitudinal designs to verify hypothesized causal pathways, utilizing Directed Acyclic Graphs (DAGs) to explicitly map confounding structures and causal directions.

Fourth, due to space constraints, this study did not delve into variable interactions. Future research should prioritize exploring these interactions to reveal how diverse social factors collectively shape individual behavior. Particularly across different socioeconomic contexts, variable interactions may yield markedly different outcomes, offering broad prospects for subsequent studies.

Fifth, specific attention must be paid to the temporal context of the data, which were collected in 2021 during the COVID-19 pandemic. While this timing offers valuable insights into behavior under the “new normal,” the observed physical exercise patterns may be partially shaped by pandemic-specific factors prevalent at that time, such as sporadic social restrictions, altered access to public facilities, or pandemic-induced health anxiety. Consequently, the behavioral mechanisms identified in this 2021 sample might reflect adaptive responses to a unique historical period rather than stable, long-term preferences. Future research is essential to verify the generalizability of these findings in the post-pandemic era and determine whether the influence of sociodemographic factors persists as society returns to full normalcy.

Furthermore, future research plans to obtain higher-quality, more comprehensive datasets. For instance, efforts will be made to secure longitudinal data spanning more extended time periods or more representative regional survey data to more effectively track changes in individual behavior and the long-term impact of social environments on such behavior. Concurrently, subsequent studies will investigate differences across regions or specific populations, further illuminating the profound influence of social factors on individual behavior. In summary, while this study offers valuable preliminary insights into understanding physical exercise participation among Chinese adults, substantial room for exploration remains. Future research will focus on enhancing data quality, optimizing research methodologies, and analyzing interactions. By integrating research findings with public policy and social practice, we aim to provide more comprehensive and precise theoretical support and practical guidance for the fields of social behavior and public health.

## Figures and Tables

**Table 1:** Predictive variables, dimensions, and references.

Theoretical Framework (SEM Level)	TPB Construct Mapping (Proxy)	Predictive Variable	Supporting References
Micro-system (Individual)	Perceived Behavioral Control (Capability)	Gender	(Metcalfe & Lindsey, 2020)
		Age	
		BMI	(Churilla et al., 2018)
		Educational attainment	(Lee & Kang, 2024)
		Socioeconomic status	(Eime et al., 2015)
		Sleep quality	(Cullen et al., 2019)
	Attitudes	Self-rated health	(Edelstein, 2023)
		Subjective well-being	(S. Chen et al., 2021)
		Perceived social fairness	(Sui et al., 2024)
		Perceived social class	(Yang et al., 2022)
		Smoking	(Mi-Ok et al., 2018)
		Drinking	(Barrett et al., 2023)
Meso-system (Social)	Subjective Norms	Social trust	(Stevens et al., 2018)
		Social activities	(Ren & Liu, 2022)
		Watching sports events	
Macro-system (Environment)	Perceived Behavioral Control (Facilitating Conditions)	Residential area suitable for physical exercise	(Prince et al., 2022)
		Residential area with sufficient public facilities	
		Residential area is very safe	(Elshahat et al., 2020)
	Structural Constraints	Household registration	(Wang & Zhou, 2018)

**Table 2:** Variable selection and cleaning.

Variable		Question Code	Title of Item	Data Processing
Dependent variable	Physical exercise participation	A30_9	Over the past year, have you frequently participated in physical exercise during your free time?	Reverse-scoring process: the higher the value, the higher the frequency
Demographics	Gender	A2	Gender	Convert to a binary variable, male = 1, female = 0
	Age	A3_1	What is your date of birth?	Raw data, 2021-year of birth = age
	Educational attainment	A7a_1	Uneducated	Raw data, with a value of 1
		A7a_2	Private tutoring, Literacy classes	Raw data, with a value of 2
		A7a_3	Elementary school	Raw data, with a value of 3
		A7a_4	Junior high school	Raw data, with a value of 4
		A7a_5	Vocational high school	Merged into high school education, with a value of 5
		A7a_6	Regular high school	
		A7a_7	Technical secondary school	
		A7a_8	Higher vocational school	
		A7a_9	University college (Adult Higher Education)	Merged into bachelor’s degree, with a value of 6.
		A7a_10	University college (formal higher education)	
		A7a_11	Bachelor’s degree (adult higher education)	
		A7a_12	Bachelor’s degree (formal higher education)	
		A7a_13	Graduate degree or above	Raw data, with a value of 7
	Household registration	A18_1	Your current household registration status is agricultural household registration.	Raw data, with a value of 1
		A18_2	Your current household registration status is non-agricultural.	Merged into resident household registration, with a value of 0
		A18_3	Your current household registration status is resident household registration (previously agricultural household registration).	
		A18_4	Your current household registration status is resident household registration (previously non-agricultural household registration).	
		A18_5	Military registration	Too few cases, defined as missing values
		A18_6	Other	
		A18_7	No household registration	
	Height	A13	Your current height is ____ centimeters	Raw data/100, convert height to meters
	Weight	A14	Your current weight is ____ 1/2 kg	Raw data/2, convert weight to kilograms
	BMI	/	/	(Height A13)/(Weight A142)
	Socioeconomic status	A43e	Overall, in today’s society, your socioeconomic status is:	Reverse scoring: the higher the value, the higher the socioeconomic status.
Subjective perception	Self-assessment of health	A15	How would you rate your current physical health?	Raw data Likert 5-point scale, ranging from very unhealthy to very healthy
	Social trust	A33	In general, do you agree that the vast majority of people in society are trustworthy?	Raw data Likert 5-point scale, ranging from Strongly Disagree to Strongly Agree
	Perceiving social fairness	A35	Overall, do you think society today is fair?	Raw data Likert 5-point scale, ranging from completely unfair to completely fair
	Subjective well-being	A36	Overall, would you say you are happy with your life?	Raw data Likert 5-point scale, ranging from very unhappy to very happy
	Social class perception	A43a	Overall, where do you think you fit into society at present?	Raw data Likert 10-point scale, where higher scores indicate a higher perceived social class
Lifestyle	Watch sports events	A30_10	Over the past year, have you often watched live sports events in your spare time?	Reverse-scoring process: the higher the value, the higher the frequency
	Social activity	A31_1	Over the past year, have you frequently socialized or visited friends and family in your spare time?	Raw data
	Smoking	E18	Do you smoke?	Reverse scoring: 1 means almost never/never smoked, 2 means used to smoke but now does not, 3 means currently smokes
	Alcohol consumption	E19	How often do you drink alcohol?	Reverse-scoring process: the higher the value, the higher the frequency
	Sleep quality	E24	How would you rate the quality of your sleep over the past month?	Reverse scoring: the higher the score, the better the sleep quality
Built environment	Residence suitable for physical exercise	E36_A	The place where I live is suitable for physical exercise, such as jogging and walking.	Reverse scoring, with higher values indicating greater agreement
	The residential area has sufficient public facilities	E36_C	There are sufficient public facilities (such as community centers, libraries, parks, etc.) where I live.	Reverse scoring, with higher values indicating greater agreement
	The neighborhood is very safe	E36_D	The place where I live is very safe.	Reverse scoring, with higher values indicating greater agreement

**Table 3:** Comparison of demographic and key characteristics between included analytic sample and excluded respondents.

Variable	*n*_Inc	*n*_Exc	Included_Desc	Excluded_Desc	Test_Stat	*p*_Value
Physical exercise participation	1563	6571	2.90 ± 1.51	2.82 ± 1.64	*t* = 1.95	0.051
Gender	1563	6585	(*N* = 1563)	(*N* = 6585)	*X*^2^ = 1.18	0.277
Age	1563	6585	41.12 ± 12.12	54.14 ± 17.74	*t* = −34.60	<0.001 ***
Educational attainment	1563	6564	(*N* = 1563)	(*N* = 6564)	*X*^2^ = 210.64	<0.001 ***
Household registration	1563	6492	(*N* = 1563)	(*N* = 6492)	*X*^2^ = 0.33	0.563
BMI	1563	6405	23.13 ± 3.63	23.03 ± 3.67	*t* = 1.01	0.314
Socioeconomic status	1563	6406	7.31 ± 0.86	7.27 ± 0.91	*t* = 1.63	0.102
Self-assessment of health	1563	6579	3.70 ± 1.00	3.43 ± 1.11	*t* = 9.51	<0.001 ***
Social trust	1563	6516	3.58 ± 0.99	3.66 ± 1.00	*t* = −2.65	0.008 **
Perceiving social fairness	1563	6527	3.38 ± 0.95	3.48 ± 0.98	*t* = −3.54	<0.001 ***
Subjective well-being	1563	3885	3.95 ± 0.79	4.00 ± 0.83	*t* = −2.14	0.033 *
Watch sports events	1563	6550	1.36 ± 0.73	1.28 ± 0.68	*t* = 3.93	<0.001 ***
Social events	1563	6579	2.69 ± 1.04	2.62 ± 1.14	*t* = 2.35	0.019 *
Smoking	1563	1154	(*N* = 1563)	(*N* = 1154)	*X*^2^ = 68.48	<0.001 ***
Alcohol consumption	1563	1154	1.83 ± 1.13	1.80 ± 1.37	*t* = 0.63	0.526
Sleep quality	1563	1154	3.92 ± 0.73	3.86 ± 0.85	*t* = 1.89	0.058
Residence suitable for physical exercise	1563	1154	3.86 ± 0.97	3.76 ± 1.07	*t* = 2.34	0.019 *
The residential area has sufficient public facilities	1563	1154	3.26 ± 1.22	3.08 ± 1.28	*t* = 3.68	<0.001 ***
The neighborhood is very safe	1563	1154	4.14 ± 0.71	4.27 ± 0.71	*t* = −4.67	<0.001 ***

Note: Data are presented as the mean ± SD for continuous variables and frequency (*n*) for categorical variables. *n*_Inc: The number of participants in the included analytic sample. *n*_Exc: The number of participants in the excluded group. *** *p* < 0.001, ** *p* < 0.01, and * *p* < 0.05.

**Table 4:** Sensitivity analysis of all 19 predictors.

Variable Category/Name	Lasso Status	Bivariate Analysis *OR (95%CI)*	*p*	Sensitivity Analysis *OR (95%CI)*	*p*
Variables Selected by Lasso (*n* = 9)					
Educational attainment	Selected	1.41 (1.30, 1.53)	*p* < 0.001	1.38 (1.23, 1.54)	*p* < 0.001
Watch sports events	Selected	1.67 (1.44, 1.93)	*p* < 0.001	1.56 (1.34, 1.83)	*p* < 0.001
Subjective well-being	Selected	1.30 (1.17, 1.45)	*p* < 0.001	1.17 (1.04, 1.33)	*p* = 0.01
Sleep quality	Selected	1.19 (1.07, 1.32)	*p* < 0.001	1.13 (1.01, 1.27)	*p* = 0.04
Residence suitable for physical exercise	Selected	1.30 (1.17, 1.44)	*p* < 0.001	1.19 (1.05, 1.34)	*p* = 0.01
Smoking	Selected	0.87 (0.77, 0.99)	*p* = 0.03	0.82 (0.70, 0.97)	*p* = 0.02
Age	Selected	0.93 (0.84, 1.02)	*p* = 0.14	1.27 (1.11, 1.45)	*p* < 0.001
Social activity	Selected	1.17 (1.06, 1.29)	*p* < 0.001	1.09 (0.98, 1.21)	*p* = 0.13
Household registration	Selected	0.57 (0.46, 0.70)	*p* < 0.001	0.84 (0.65, 1.07)	*p* = 0.16
Variables Excluded by Lasso (*n* = 10)					
Gender	Excluded	1.12 (0.91, 1.37)	*p* = 0.28	1.07 (0.81, 1.41)	*p* = 0.63
BMI	Excluded	0.94 (0.85, 1.04)	*p* = 0.23	0.96 (0.85, 1.07)	*p* = 0.45
Self-assessment of health	Excluded	1.18 (1.07, 1.31)	*p* < 0.001	1.04 (0.91, 1.18)	*p* = 0.58
Social trust	Excluded	1.11 (1.00, 1.22)	*p* = 0.05	1.01 (0.90, 1.13)	*p* = 0.91
Perceiving social fairness	Excluded	1.06 (0.96, 1.17)	*p* = 0.26	0.95 (0.85, 1.07)	*p* = 0.40
Social class perception	Excluded	1.19 (1.07, 1.32)	*p* < 0.001	1.01 (0.88, 1.15)	*p* = 0.88
Socioeconomic status	Excluded	1.16 (1.05, 1.29)	*p* < 0.001	0.98 (0.86, 1.12)	*p* = 0.81
Alcohol consumption	Excluded	1.06 (0.97, 1.16)	*p* = 0.22	1.04 (0.93, 1.17)	*p* = 0.47
The residential area has sufficient public facilities	Excluded	1.24 (1.12, 1.37)	*p* < 0.001	1.03 (0.91, 1.16)	*p* = 0.68
The neighborhood is very safe	Excluded	1.09 (0.98, 1.20)	*p* = 0.11	1.03 (0.92, 1.16)	*p* = 0.59

**Table 5:** Performance of machine learning models for predicting physical exercise participation.

Model	Sensitivity *(95%CI)*	Specificity *(95%CI)*	F1 Score *(95%CI)*	Recall *(95%CI)*	Accuracy *(95%CI)*	Threshold
LR (Default)	0.38 (0.30–0.46)	0.84 (0.79–0.89)	0.47 (0.38–0.55)	0.38 (0.30–0.46)	0.65 (0.60–0.70)	0.50
LR (Optimized)	0.78 (0.71–0.85)	0.51 (0.44–0.59)	0.63 (0.56–0.69)	0.78 (0.71–0.85)	0.62 (0.57–0.68)	0.39
SVM (Default)	0.34 (0.26–0.42)	0.84 (0.79–0.89)	0.44 (0.35–0.52)	0.34 (0.26–0.42)	0.64 (0.59–0.69)	0.50
SVM (Optimized)	0.64 (0.56–0.72)	0.66 (0.59–0.73)	0.60 (0.53–0.67)	0.64 (0.56–0.72)	0.65 (0.60–0.71)	0.37
XGBoost (Default)	0.34 (0.30–0.47)	0.78 (0.72–0.84)	0.45 (0.37–0.53)	0.38 (0.30–0.47)	0.62 (0.57–0.67)	0.50
XGBoost (Optimized)	0.72 (0.64–0.80)	0.52 (0.45–0.59)	0.60 (0.53–0.66)	0.72 (0.64–0.80)	0.60 (0.55–0.66)	0.36
MLP (Default)	0.43 (0.34–0.52)	0.80 (0.73–0.85)	0.50 (0.41–0.58)	0.43 (0.34–0.52)	0.65 (0.59–0.70)	0.50
MLP (Optimized)	0.53 (0.44–0.61)	0.72 (0.65–0.78)	0.55 (0.47–0.62)	0.53 (0.44–0.61)	0.64 (0.59–0.69)	0.46
LGBM (Default)	0.42 (0.35–0.52)	0.72 (0.66–0.79)	0.48 (0.39–0.55)	0.44 (0.35–0.52)	0.61 (0.56–0.66)	0.50
LGBM (Optimized)	0.52 (0.43–0.60)	0.68 (0.61–0.75)	0.52 (0.44–0.59)	0.52 (0.43–0.60)	0.61 (0.56–0.67)	0.41

Note: Metrics presented above are based on optimized thresholds using Youden’s Index. SVM model was selected as final model due to its balanced sensitivity and specificity profile compared to baseline.

**Table 6:** Descriptive statistics of predictors retained in SVM model.

Variable	Mean ± SD	Min–Max	Median [Q1, Q3]	IQR
Age (years)	41.12 ± 12.12	18–59	42 [32, 52]	20
Educational attainment	4.55 ± 1.33	1–7	5 [4, 6]	2
Household registration (0/1)	0.59 ±0.49	0–1	1 [0, 1]	1
Watching sports events (1–5)	1.36 ± 0.73	1–5	1 [1, 1]	0
Social activities (1–5)	2.69 ± 1.04	1–5	3 [2, 3]	1
Subjective well-being (1–5)	3.95 ± 0.79	1–5	4 [4, 4]	0
Smoking status (1–3)	1.50 ± 0.83	1–3	1 [1, 2]	1
Sleep quality (1–4)	2.92 ± 0.73	1–4	3 [3, 3]	0
Residence suitable for physical exercise (1–5)	3.86 ± 0.97	1–5	4 [4, 4]	0

## Data Availability

The data presented in this study are openly available in the Chinese National Survey Data Archive repository at http://www.cnsda.org/index.php?r=projects/view&id=65635422 (accessed on 2 July 2025).
